# Endotoxemia-induced cytokine-mediated responses of hippocampal astrocytes transmitted by cells of the brain–immune interface

**DOI:** 10.1038/srep25457

**Published:** 2016-05-05

**Authors:** Sanae Hasegawa-Ishii, Muneo Inaba, Hiroyuki Umegaki, Keiko Unno, Keiji Wakabayashi, Atsuyoshi Shimada

**Affiliations:** 1 Japan Society for the Promotion of Science, 5-3-1 Kojimachi, Chiyoda, Tokyo 102-0083, Japan; 2 Department of Pathology and Laboratory Medicine, Central Hospital, Aichi Human Service Center, 713-8 Kamiya, Kasugai, Aichi 480-0392, Japan; 3 Graduate school of Integrated Pharmaceutical and Nutritional Sciences, University of Shizuoka, 52-1 Yada, Suruga-ku, Shizuoka 422-8526, Japan; 4 First Department of Internal Medicine, Kansai Medical University, 2-5-1 Shinmachi, Hirakata, Osaka 573-1010, Japan; 5 Department of Geriatrics, Graduate School of Medicine, Nagoya University, 65 Tsurumai, Showa-ku, Nagoya, Aichi 466-8550, Japan; 6 Department of Neurophysiology, School of Pharmaceutical Sciences, University of Shizuoka, 52-1 Yada, Suruga-ku, Shizuoka 422-8526, Japan

## Abstract

Systemic inflammation shifts the brain microenvironment towards a proinflammatory state. However, how peripheral inflammation mediates changes in the brain remains to be clarified. We aimed to identify hippocampal cells and cytokines that respond to endotoxemia. Mice were intraperitoneally injected with lipopolysaccharide (LPS) or saline, and examined 1, 4, and 24 h after injection. Tissue cytokine concentrations in the spleens and hippocampi were determined by multiplex assays. Another group of mice were studied immunohistologically. Fourteen cytokines showed an increased concentration in the spleen, and 10 showed an increase in the hippocampus after LPS injection. Cytokines increased at 4 h (CCL2, CXCL1, CXCL2, and interleukin-6) were expressed by leptomeningeal stromal cells, choroid plexus stromal cells, choroid plexus epithelial cells, and hippocampal vascular endothelial cells, all of which were located at the brain–immune interface. Receptors for these cytokines were expressed by astrocytic endfeet. Cytokines increased at 24 h (CCL11, CXCL10, and granulocyte-colony stimulating factor) were expressed by astrocytes. Cells of the brain–immune interface therefore respond to endotoxemia with cytokine signals earlier than hippocampal parenchymal cells. In the parenchyma, astrocytes play a key role in responding to signals by using endfeet located in close apposition to the interface cells via cytokine receptors.

Determining the mechanism underlying bi-directional communication between the central nervous system (CNS) and the immune system is challenging. Although sympathetic activities regulate local immune responses^1^, systemic inflammation shifts the brain microenvironment towards a proinflammatory state, leading to behavioral alterations such as sickness behavior^2^.

The hippocampus is one of the targets for behavioral changes induced by systemic inflammatory challenge. Forms of memory sensitive to peripheral administration of lipopolysaccharide (LPS), such as the spatial water maze task^3^ and contextual fear conditioning^4^, tend to be hippocampal dependent. Peripherally administered LPS impairs context discrimination memory by disrupting specific neural circuits within the hippocampus^5^. However, LPS does not cross the blood–brain barrier (BBB)^6^. The amount of LPS entering brain parenchyma is only about 0.025% of an intravenously administered dose, which suggests that most effects induced by acute peripheral administration of LPS are not mediated through receptors expressed by brain parenchymal cells^7^. Thus, how LPS outside the BBB mediates changes inside the brain remains uncertain.

Clinically, excessive systemic inflammatory reaction to an infection may induce sepsis-associated encephalopathy (SAE). Some septic patients develop diffuse brain dysfunction such as delirium, cognitive impairments, loss of consciousness, and sometimes even epileptic seizures^8^. SAE is different from encephalitis and meningitis, which are associated with bacterial invasion into the brain and meninges, respectively. In SAE, bacteria are not found in the nervous system but affect brain function, indicating that SAE is not caused by the direct infection of the brain with microorganisms but is based on a different mechanism^9^,^10^.

Systemic administration of bacteria, LPS, or proinflammatory cytokines such as interleukin (IL)-1β and tumor necrosis factor (TNF)-α triggers rapid transcriptional activation of genes encoding various proinflammatory molecules, including IL-1β^11^,^12^,^13^, IL-6^14^, TNF-α^14^, C-C motif ligand (CCL)2^15^, cyclooxygenase-2^16^, and CD14^17^ in the choroid plexus, leptomeninges, circumventricular organs (CVOs), and cerebral blood vessels. More recent studies using multiplex assays following systemic administration of LPS revealed that concentrations of a variety of cytokines increase in the brain parenchyma and in serum^18^,^19^. Based on these studies, some intercommunication between the CNS and immune system in response to endotoxemia has been suggested. Four possible routes by which the CNS and immune system interact with each other have been proposed^20^: a neural route via sympathetic or vagus nerves^21^, CVOs, transport by cellular components that form the BBB^22^, and secretion by vascular endothelial cells^22^.

Although knowledge about the brain–immune communication pathway is accumulating, particular cells of the hippocampus and related structures that produce mediator cytokines in response to systemic inflammation remain to be determined. Clarification of how hippocampal parenchymal cells respond to cytokine-mediated signals during the acute phase of systemic inflammation is relevant to the understanding of hippocampal impairments induced by systemic immune activation.

In this study, we used Luminex multiplex assay technology to determine cytokines that show a change in concentration in the hippocampus and spleen in response to systemic administration of LPS. We then immunohistologically identified cells involved in the elevation of hippocampal cytokine levels.

## Results

### Changes in the tissue cytokine concentrations after systemic LPS challenge

Based on the results of one-way ANOVA and post hoc tests with Tukey’s procedure that indicated a significant difference or trend towards significance (0.05 ≤ *p* < 0.06) between experimental conditions, time-dependent changes in the tissue cytokine concentrations were compiled into six patterns (Table 1).

**Figure 1:**
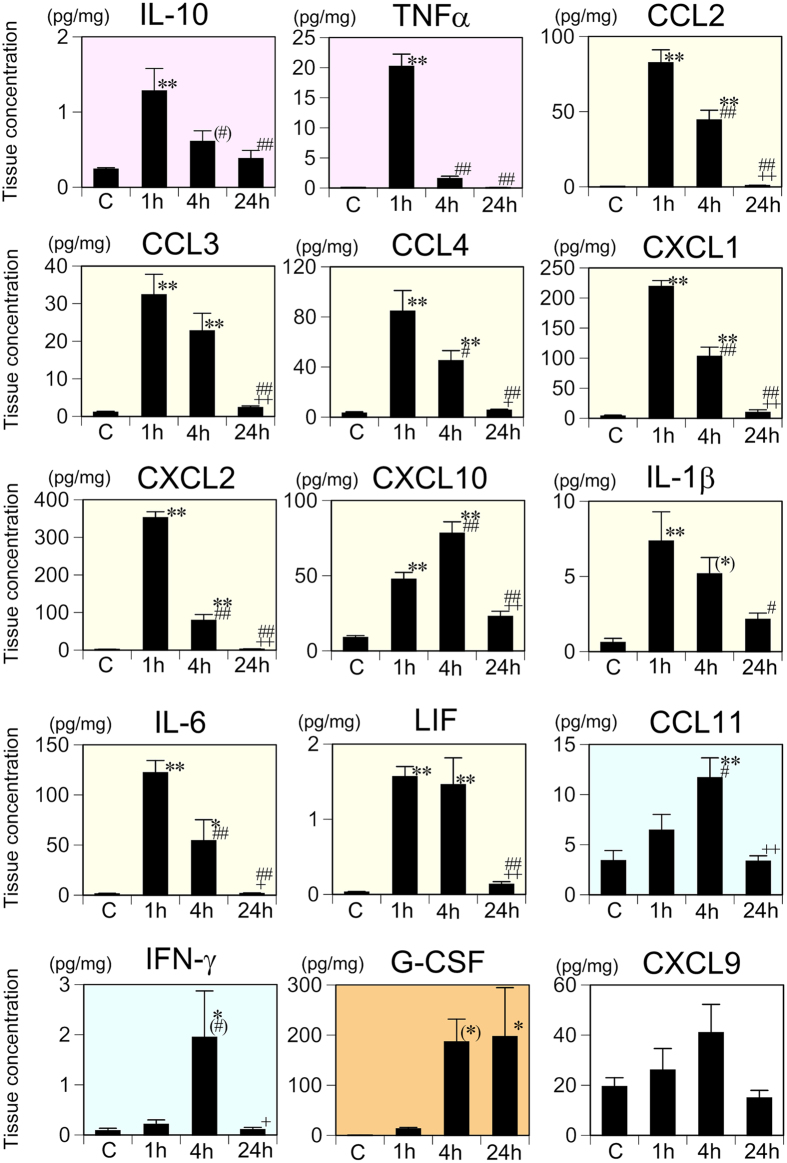
Changes in the splenic tissue concentrations of 15 cytokines following systemic LPS challenge. Cytokine concentrations were measured with the multiplex assay and comparisons made among the saline control (C) and 1, 4, and 24 h following systemic LPS injection. Data were analyzed with one-way ANOVA followed by Tukey’s post hoc procedure. All cytokines were categorized into one of groups A–F according to the patterns of time-dependent changes in the tissue concentration (Table 3). Graphs for the group A cytokines are indicated with a *pink* background, group B with *yellow*, group C with *blue*, group D with *orange*, and group F with *white*. Mean ± SEM. For CCL2, IL-1β, and LIF, *n* = 5 per experimental group and for the other 12 cytokines, *n* = 7 for saline control and *n* = 5 for each LPS treatment condition. **p < 0.01, *p < 0.05, and (*)0.05 ≤ *p* < 0.06, compared with the saline control; ^##^*p* < 0.01, ^#^*p* < 0.05, and ^(#)^0.05 ≤ *p* < 0.06, compared with 1 h after LPS injection;^++^*p* < 0.01 and ^+^*p* < 0.05, compared with 4 h after LPS injection.

In the spleen, one-way ANOVA revealed that tissue concentrations of 14 of the 15 cytokines examined (the exception being CXCL9) increased significantly after LPS injection (Supplementary Table 1). According to the criteria as described in Table 1, IL-10 and TNF-α were categorized as group A; CCL2, CCL3, CCL4, CXCL1, CXCL2, CXCL10, IL-1β, IL-6, and LIF as group B; CCL11 and IFN-γ as group C; G-CSF as group D; and CXCL9 as group F (Fig. 1).

**Figure 2:**
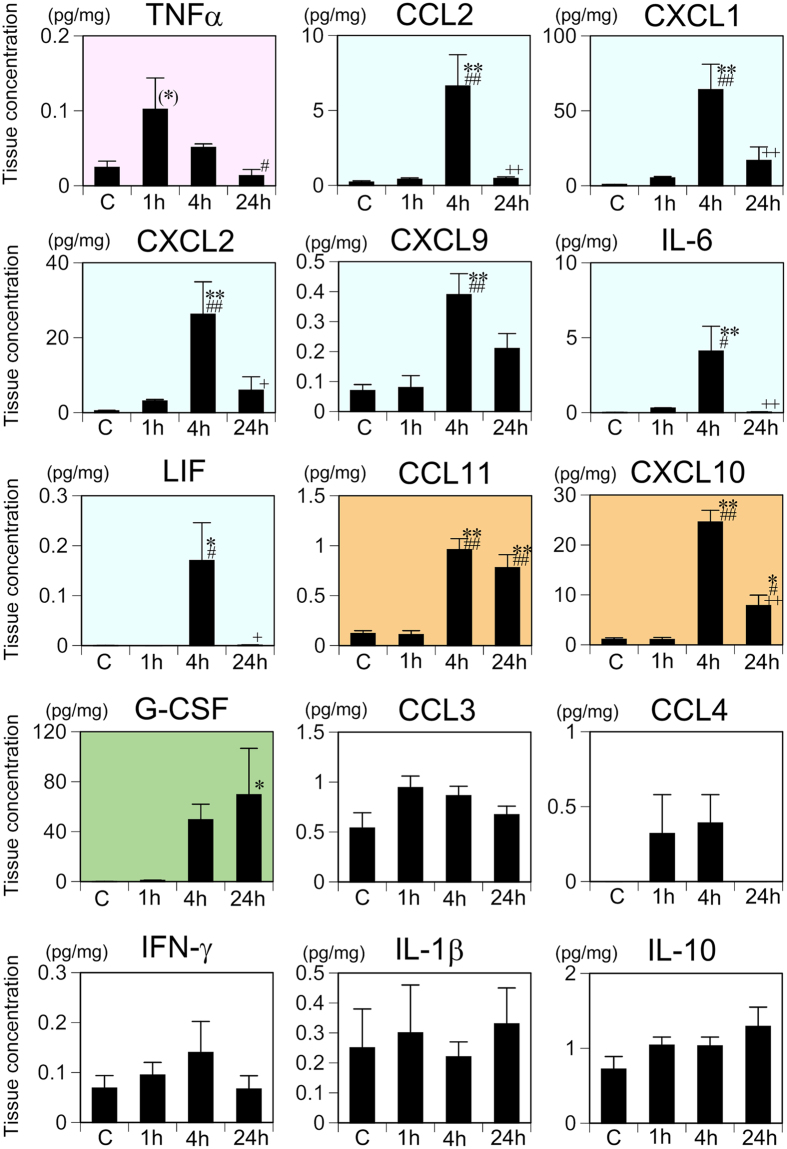
Changes in the hippocampal tissue concentrations of 15 cytokines following systemic LPS challenge. Cytokine concentrations were measured with the multiplex assay and comparisons made among the saline control (C) and 1, 4, and 24 h following systemic LPS injection. Data were analyzed with one-way ANOVA followed by Tukey’s post hoc procedure. All cytokines were categorized into one of groups A–F according to the patterns of time-dependent changes in the tissue concentration. Graphs for the group A cytokines are indicated with a *pink* background, group C with *blue*, group D with *orange*, group E with *green*, and group F with *white*. Mean ± SEM. For CCL2, IL-1β, and LIF, *n* = 5 per experimental condition and for the other 12 cytokines, *n* = 7 for saline control and *n* = 5 for each LPS treatment condition. **p < 0.01, *p < 0.05, and (*)0.05 ≤ *p* < 0.06, compared with the saline control; ^##^*p* < 0.01 and ^#^*p* < 0.05, compared with 1 h after LPS injection; ^++^*p* < 0.01 and ^+^*p* < 0.05, compared with 4 h after LPS injection.

In the hippocampus, one-way ANOVA revealed that tissue concentrations of 10 of the 15 cytokines increased significantly after LPS injection (Supplementary Table 2). According to the criteria as described in Table 1, TNF-α was categorized as group A; CCL2, CXCL1, CXCL2, CXCL9, IL-6, and LIF as group C; CCL11 and CXCL10 as group D; G-CSF as group E; and CCL3, CCL4, IFN-γ, IL-1β, and IL-10 as group F (Fig. 2). There was a general trend that the increase in tissue cytokine concentration occurred earlier in the spleen than in the hippocampus.

### Immunohistological identification of hippocampal cells expressing cytokines that were increased at 4 h but not 24 h after LPS injection

**Figure 3:**
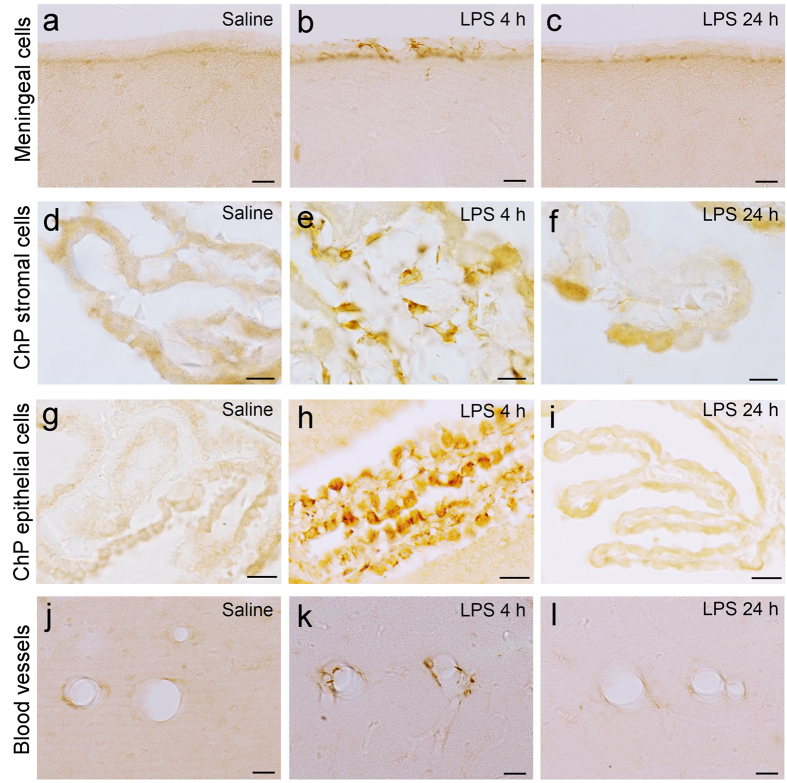
CCL2 expression in the hippocampus following systemic LPS challenge. Immunohistochemistry for CCL2 revealed that CCL2 expression was not detected in the saline control in any region of the hippocampus (**a,d,g,j**) and was increased in the leptomeninges (**b**), choroid plexus stroma (**e**), and choroid plexus epithelial cells (**h**) and along the hippocampal blood vessels (**k**) 4 h after LPS injection. CCL2 expression returned to the control appearance in the hippocampus 24 h after LPS injection (**c**,**f**,**i**,**l**). *Scale bars* (**a–c,g–l**) 20 μm; (**d**–**f**) 10 μm. *ChP* choroid plexus.

We performed immunohistological staining for the 10 cytokines that were shown to be increased in the hippocampus after LPS injection by multiplex assays to identify cellular and tissue components contributing to the elevation of hippocampal cytokine concentrations. Among the hippocampal group C cytokines, CCL2 expression increased in specific areas of the hippocampus at 4 h after LPS injection compared with the saline control (Fig. 3). CCL2 was expressed in some cells of leptomeninges (Fig. 3b) and choroid plexus stroma (Fig. 3e), which exhibited a spindle shape with multiple thin processes (suggestive of reticular cells), and in the choroid plexus epithelial cells (Fig. 3h). CCL2 was also expressed along the blood vessels of the hippocampal parenchyma (Fig. 3k). CCL2 expression in the hippocampus returned to the control appearance 24 h after LPS injection (Fig. 3c,f,i,l).

Leptomeninges and choroid plexus stroma share tissue components including endothelial cells, pericytes, myeloid cells^23^,^24^, arachnoid cells, fibroblasts, collagen, and extracellular matrix^25^, among others. Our double immunofluorescence staining revealed that most of the CCL2-expressing cells in the leptomeninges and choroid plexus stroma were immunonegative for cell markers such as CD31 (endothelial cells), NG2 (pericytes), Iba-1 (myeloid cells), S100 (arachnoid cells), or ER-TR7 (fibroblasts), although a minor population of CCL2-expressing cells was immunopositive for Iba-1 and ER-TR7 (Supplementary Fig. 1). Regarding NG2-immunopositivity, NG2 has been known to be expressed by oligodendrocyte progenitor cells in the brain parenchyma. By contrast, in the leptomeninges and choroid plexus stroma, NG2-immunopositive cells were in close apposition to CD31-immunopositive endothelial cells. Thus, we were able to identify these NG2-immunopositive cells as pericytes. Because CCL2-expressing cells were not endothelial cells, pericytes, myeloid cells, arachnoid cells, or fibroblasts, we simply express these cells as “stromal cells.” Similarly to the hippocampus, CCL2-immunoreactive cells in the spleen were immunonegative for CD31, NG2, Iba-1, and ER-TR7 in mice 1 h after LPS injection (Supplementary Fig. 2).

The identification of cells along the hippocampal parenchymal blood vessels that expressed CCL2 was not successful by double immunofluorescence staining for CCL2 and CD31. Colocalization analysis indicated that the Pearson’s correlation coefficient ranged from 0.20 to 0.38 with the mean being 0.38 (5 fields), which was interpreted as a low correlation between CCL2- and CD31-immunofluorescence.

The patterns of expression of CXCL1 and CXCL2 (hippocampal group C cytokines) were similar, which is probably associated with the fact that protein sequences of mouse CXCL1 and CXCL2 exhibit approximately 63% homology. Colocalization analysis following double immunofluorescence staining for CXCL1 and CXCL2 indicated that the Pearson’s correlation coefficient ranged from 0.861 to 0.988 with the mean being 0.943 (8 fields), which was interpreted as a very high correlation between CXCL1- and CXCL2-immunofluorescence. The expression of CXCL1 and CXCL2 increased at 4 h after LPS injection compared with saline control along hippocampal blood vessels, in choroid plexus epithelial cells, and in some cells of the leptomeninges and choroid plexus stroma that exhibited a spindle shape with multiple processes (Supplementary Fig. 3a,b,d,e,g). The expression returned to the control appearance 24 h after LPS injection (Supplementary Fig. 3c,f). Double immunofluorescence staining for CXCL1 and CD31revealed that CXCL1-immunopositive cells along hippocampal blood vessels after LPS injection were endothelial cells, as evidenced by immunopositivity for CD31 (Supplementary Fig. 3g–i). Colocalization analysis indicated that the Pearson’s correlation coefficient ranged from 0.411 to 0.946 with the mean being 0.683 (8 fields), which was interpreted as a moderate correlation between CXCL1- and CD31-immunofluorescence. By contrast, colocalization analysis following double immunofluorescence staining for CXCL2 and CD31 indicated that the Pearson’s correlation coefficient ranged variably from 0.153 to 0.946 with the mean being 0.411 (8 fields), which was interpreted as a low correlation. The major portion of CXCL1-immunopositive cells in the leptomeninges and choroid plexus stroma were immunonegative for CD31, NG2, Iba-1, S100, and ER-TR7 and, therefore, “stromal cells.”

Cells expressing IL-6, another hippocampal group C cytokine, appeared only in the leptomeninges and choroid plexus stroma at 4 h after LPS injection and exhibited a spindle shape with thin processes, although IL-6 was not detected in any region of the hippocampus in saline control (Supplementary Fig. 4). IL-6 expression returned to the control appearance 24 h after LPS injection. Double immunofluorescence staining revealed that the major populations of IL-6-immunopositive cells in the choroid plexus stroma were immunonegative for CD31, NG2, Iba-1, S100, and ER-TR7 and, therefore, “stromal cells.”

CCL2, CXCL1, CXCL2 or IL-6 was not expressed in the hippocampal neurons or glial cells. For example, colocalization analysis following double immunofluorescence staining for CCL2 and GFAP indicated that the Pearson’s correlation coefficient ranged from 0.028 to 0.354 with the mean being 0.118 (9 fields), which was interpreted as a negligible correlation.

### Immunohistological identification of hippocampal cells expressing cytokines that were increased at 24 h after LPS injection

**Figure 4:**
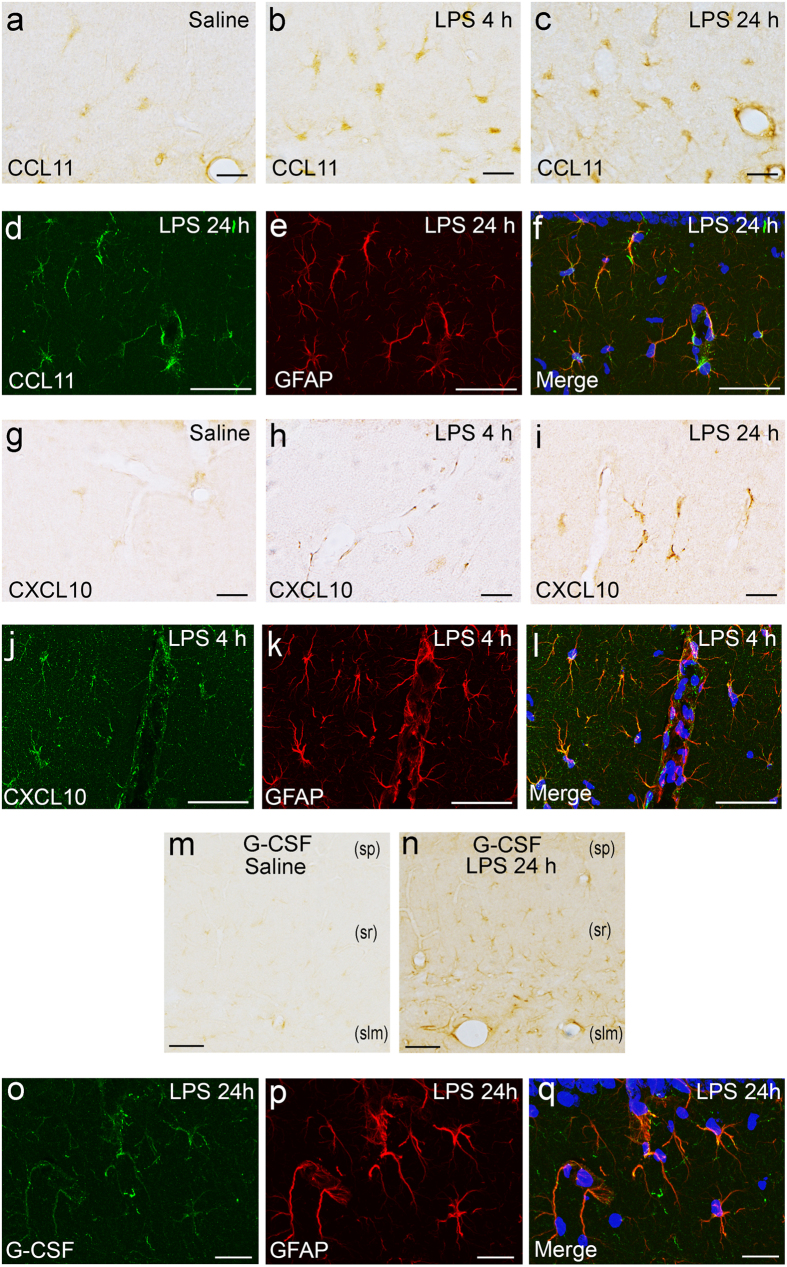
Identification of CCL11-, CXCL10-, and G-CSF-producing cells in the hippocampus following systemic LPS challenge. Immunohistochemistry for CCL11 revealed that CCL11 was expressed weakly in the hippocampal parenchyma of the saline control (**a**) and was increased in hippocampal glial cells at 4 h (**b**) and 24 h (**c**) after LPS injection. Double immunofluorescence study revealed that CCL11-expressing cells in the hippocampal parenchyma were immunopositive for GFAP, indicative of astrocytes (**d**–**f**). Immunohistochemistry for CXCL10 revealed that CXCL10 was expressed weakly in some glial cells in the hippocampal parenchyma of the saline control (**g**), and was increased along the blood vessels (**h**) and in glial cells in the hippocampal parenchyma (**i**) at 4 and 24 h after LPS injection. Double immunofluorescence study revealed that CXCL10-expressing cells were immunopositive for GFAP, indicative of astrocytes (**j**–**l**). Immunohistochemistry revealed that G-CSF expression was not detected in the saline control (**m**) and was increased in the hippocampal parenchyma 24 h after LPS injection (**n**). (*sp)* stratum pyramidale; (*sr)* stratum radiatum; (*slm)* stratum lacunosum-moleculare. Double immunofluorescence study revealed that G-CSF-expressing cells in the hippocampal parenchyma were immunopositive for GFAP, indicative of astrocytes (**o**–**q**). Photoimages of (**d**–**f**, **j**–**l**), and (**o**–**q**) were obtained from mice at 24 h, 4 h, and 24 h after LPS injection, respectively. Nuclei were counterstained with DAPI in immunofluorescence staining. *Scale bars* (**a**–**c, g-i, o**–**q**) 20 μm; (**d**–**f**, **j**–**n**) 50 μm.

Among the hippocampal group D cytokines, CCL11 was expressed in cytoplasmic processes of glial cells in the strata lacunosum-moleculare, radiatum, and oriens, and in the alveus of hippocampus at 4 and 24 h after LPS injection (Fig. 4a–c). Double immunofluorescence staining for CCL11 and GFAP revealed that CCL11-immunopositive glial cells were immunopositive for glial fibrillary acidic protein (GFAP), which is indicative of astrocytes (Fig. 4d–f). Colocalization analysis indicated that the Pearson’s correlation coefficient ranged from 0.718 to 0.895 with the mean being 0.809 (10 fields), which was interpreted as a high correlation between CCL11- and GFAP-immunofluorescence.

The expression of CXCL10, the other hippocampal group D cytokine, increased along hippocampal blood vessels (Fig. 4e), in some glial cells of the hippocampal parenchyma (Fig. 4f), and in spindle-shaped cells of the leptomeninges and choroid plexus stroma at 4 and 24 h after LPS injection compared with saline control (Fig. 4d). Double immunofluorescence staining revealed that CXCL10-immunopositive glial cells were GFAP-immunopositive astrocytes (Fig. 4g–i). Colocalization analysis indicated that the Pearson’s correlation coefficient ranged from 0.486 to 0.732 with the mean being 0.649 (5 fields), which was interpreted as a moderate correlation between CXCL10- and GFAP-immunofluorescence.

G-CSF, a hippocampal group E cytokine, was expressed in glial cells of the hippocampus at 24 h after LPS injection (Fig. 4n), although it was not expressed in any region of the hippocampus in saline control (Fig. 4m). Double immunofluorescence staining for G-CSF and GFAP revealed that the major population of hippocampal G-CSF-immunopositive cells was immunopositive for GFAP, indicating astrocytes (Fig. 4o–q). Colocalization analysis indicated that the Pearson’s correlation coefficient ranged from 0.639 to 0.898 with the mean being 0.805 (10 fields), which was interpreted as a high correlation between G-CSF- and GFAP-immunofluorescence.

### Immunohistological identification of hippocampal cells expressing receptors for cytokines

**Figure 5:**
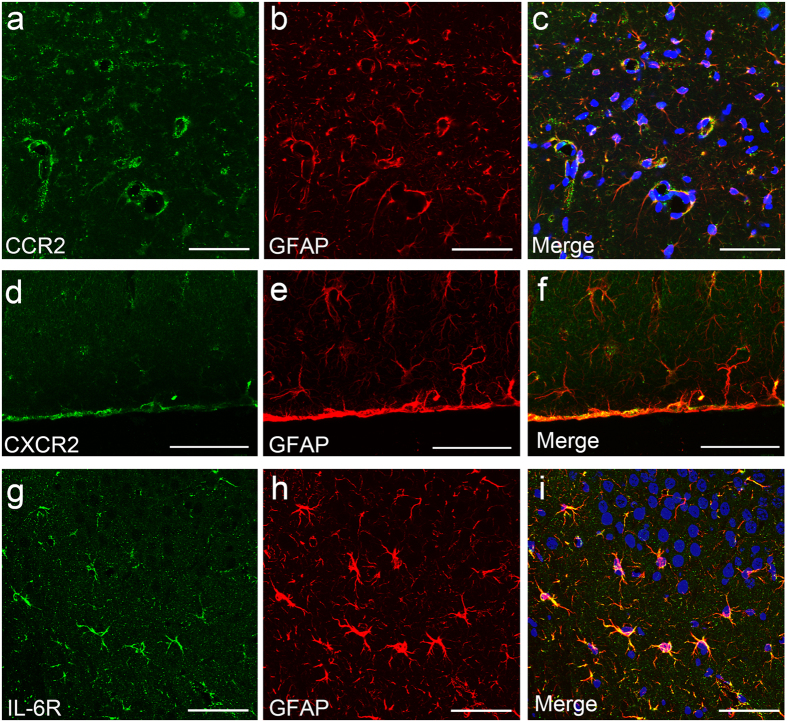
Identification of receptor-expressing cells in the hippocampus following systemic LPS challenge. Double immunofluorescence study revealed that CCR2-expressing cells in the hippocampal parenchyma were immunopositive for GFAP, indicative of astrocytes (**a**–**c**). CCR2 expression was especially pronounced in astrocytic endfeet surrounding blood vessels. CXCR2-expressing cells in the hippocampal parenchyma were immunopositive for GFAP, indicative of astrocytes (**d**–**f**). CXCR2 expression was especially pronounced just underneath the pia (**d**–**f**). IL-6R-expressing cells in the hippocampal parenchyma were immunopositive for GFAP, indicative of astrocytes (**g**–**i**). Nuclei were counterstained with DAPI. All photoimages were obtained from LPS-treated mice. *Scale bars* 50 μm.

Double immunofluorescence staining for CCR2, a receptor for CCL2 and GFAP revealed that CCR2 was expressed in GFAP-immunopositive astrocytes in the hippocampal parenchyma (Fig. 5a–c). CCR2 expression was especially pronounced in astrocytic endfeet surrounding blood vessels. Colocalization analysis indicated that the Pearson’s correlation coefficient ranged from 0.709 to 0.933 with the mean being 0.826 (10 fields), which was interpreted as a high correlation between CCR2- and GFAP-immunofluorescence.

Double immunofluorescence staining for CXCR2, a common receptor for CXCL1 and CXCL2 and GFAP revealed that CXCR2 was expressed in GFAP-immunopositive astrocytes, particularly in the endfeet surrounding blood vessels and just underneath the pia covering the brain surface (Fig. 5d–f). Colocalization analysis indicated that the Pearson’s correlation coefficient ranged from 0.569 to 0.938 with the mean being 0.784 (6 fields), which was interpreted as a high correlation between CXCR2- and GFAP-immunofluorescence.

Double immunofluorescence staining for IL-6R, a receptor for IL-6 and GFAP revealed that IL-6R was expressed in GFAP-immunopositive astrocytes in the hippocampal parenchyma (Fig. 5g–i). Colocalization analysis indicated that the Pearson’s correlation coefficient ranged from 0.725 to 0.964 with the mean being 0.886 (5 fields), which was interpreted as a high correlation between IL-6R- and GFAP-immunofluorescence.

Thus, all of the receptors for cytokines that were increased 4 h after LPS injection were expressed by hippocampal astrocytes, and staining was most pronounced in the endfeet.

### Cytoskeletal alterations in hippocampal astrocytes after systemic LPS challenge

**Figure 6:**
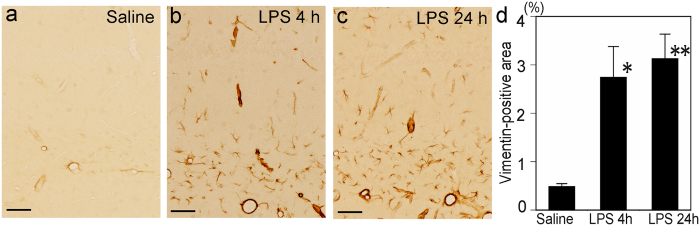
Increase in vimentin expression in the hippocampal parenchyma following systemic LPS challenge. Immunohistochemistry revealed that vimentin expression was detected only around some blood vessels in the saline control (**a**) and was increased in the hippocampal parenchyma not only around the blood vessels, but also in astrocytic cytoplasmic processes not associated with blood vessels 4 and 24 h after LPS injection (**b**,**c**, respectively). The percentage of the vimentin-immunopositive area was increased significantly 4 and 24 h after LPS injection compared with the saline control (**d**). *Scale bars* 50 μm. **p < 0.01 and *p < 0.05, compared with the saline control.

Vimentin immunoreactivity became pronounced in astrocytic endfeet, particularly in those surrounding blood vessels of the strata lacunosum-moleculare and radiatum and in the alveus, at 4 and 24 h after LPS injection compared with the saline control (Fig. 6a–c). In addition, astrocytic cytoplasmic processes that were not necessarily associated with blood vessels exhibited vimentin immunoreactivity, which was not seen in the saline control (Fig. 6a–c). The percentage of the vimentin-positive area differed significantly among experimental conditions (saline control, LPS 4 h, and LPS 24 h) [*F*(2, 12) = 9.13003, *p* = 0.003889]. Post hoc tests using Tukey’s procedure revealed that mean vimentin-immunopositive areas increased significantly in mice at 4 h (*p* = 0.014212) and 24 h (*p* = 0.005152) after LPS injection compared with saline control (Fig. 6d).

## Discussion

Ten cytokines showed an increase in concentration in the hippocampus in response to endotoxemia induced by a single i.p. injection of LPS at a dose of 3 mg/kg into mice. Leptomeningeal stromal cells, choroid plexus stromal cells, and choroid plexus epithelial cells produced CCL2, CXCL1, CXCL2, and IL-6 transiently at 4 h but not at 24 h after LPS injection. Hippocampal vascular endothelial cells produced CXCL1 at 4 h after LPS injection. Importantly, these cells are located at the brain–immune interface but not in the brain parenchyma. Furthermore, increased cytokine concentrations at 4 h after LPS injection returned to the control level, which indicates that CCL2, CXCL1, CXCL2, and IL-6 are not constitutively present in the hippocampus.

By contrast, increased cytokine concentrations in the hippocampus later than 4 h and up to 24 h after LPS injection were produced chiefly by brain parenchymal astrocytes. Based on the fact that astrocytic processes, especially endfeet, expressed the receptors for CCL2, CXCL1, CXCL2, and IL-6, we believe that astrocytes are exposed to these cytokines on endfeet that are located in close apposition to cytokine-producing cells at the brain–immune interface. Although CCL2, CXCL1, and CXCL2 are in the chemokine family, functions of these cytokines are not considered to be related to chemoattraction in this case because astrocytes are unlikely to be chemoattracted. It has been reported that astrocytes can express CCR2^26^ and CXCR2^27^ as well as IL-6R^28^, and can exert specific functions via these receptors. Activated astrocytes can release CCL11^29^ and G-CSF^30^. Therefore, the stimulation of astrocytic endfeet with cytokines released by interface cells is very likely to induce astrocytes to release another group of cytokines including CXCL10, CCL11, and G-CSF into the hippocampal interstitium. Interestingly, an increase in cytokines in the hippocampus occurred later than in the spleen. This suggests that the brain requires more steps to produce cytokines than the spleen in response to endotoxemia. We propose a novel possible route for the brain–immune interaction whereby cells in the brain–immune interface communicate with astrocytes via cytokines.

Astrocytes responded to cytokine signals, became reactive by utilizing vimentin, and took part in changing the hippocampal cytokine microenvironment earlier than other brain parenchymal cells. Vimentin is a class III intermediate filament protein that is abundantly expressed in immature astrocytes early in development. The vimentin expression disappears as astrocytes mature^31^ and reappears in reactive astrocytes after pathological insults such as repeated seizures^32^, traumatic injury^33^, and hypoxia–ischemia^34^. In the present study, we quantified the intensity of vimentin immunoreactivity as a marker for astrocytic reaction. Vimentin immunoreactivity was upregulated 4 and 24 h after LPS injection, most prominently in perivascular endfeet in the stratum lacunosum-moleculare, and to a lesser extent in astrocytes in the stratum radiatum and alveus. Surprisingly, astrocytic cytoskeletal alterations were detected as early as 4 h after systemic LPS challenge. These suggest that astrocytes reconstruct cytoskeletal components and change morphology of cytoplasmic processes so that they prepare for receiving cytokine signals via receptors and then produce their own cytokines to change hippocampal microenvironment.

Astrocytes produced CCL11 in response to endotoxemia. CCL11 is a strong eosinophil chemoattractant that mediates allergic diseases. In the brain, CCL11 is known to decrease adult neurogenesis and impair learning and memory^35^. This mechanism may underlie the hippocampal impairments induced by systemic immune activation. Another possibility is raised by a recent paper reporting that activated astrocytes release CCL11 and microglia express CCL11 receptor. CCL11 promotes the migration of microglia and induces microglia to produce reactive oxygen species by upregulating nicotinamide adenine dinucleotide phosphate-oxidase 1, thereby enhancing excitotoxic neuronal death^29^. Thus, astrocyte-derived CCL11 in our model may stimulate microglia to cause hippocampal neuronal damage, leading to behavioral changes and memory impairment.

G-CSF, by contrast, is a growth factor that stimulates proliferation, differentiation, and survival of hematopoietic progenitor cells. Many studies have indicated that G-CSF exerts neuroprotective actions on cerebral ischemic damage by promoting neuronal progenitor responses^36^,^37^. However, systemic injection of G-CSF into uninjured mice enhances the proliferation of microglia in the intact hippocampus^38^, suggesting that G-CSF may act as a growth factor for microglia. In our model, there was no evidence of cellular injury or hypoxic–ischemic brain tissue damage. Thus, astrocyte-derived G-CSF in our model may enhance the proliferation of microglia in the hippocampus, resulting in behavioral changes and memory impairment.

**Figure 7:**
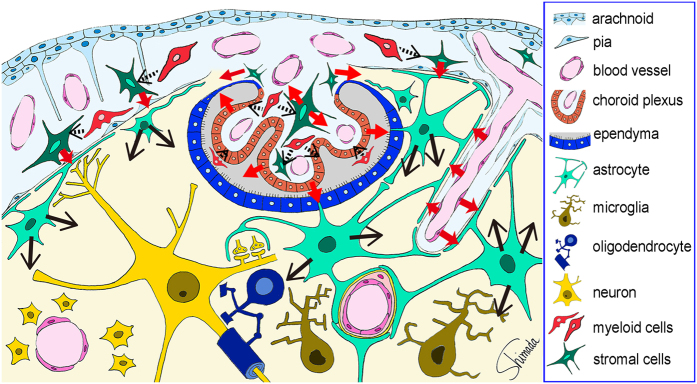
Schematic illustration of the possible cytokine flow pathway by which systemic inflammation changes the brain cytokine microenvironment. In response to the endotoxin-induced systemic inflammation, hippocampal vascular endothelial cells, leptomeningeal stromal cells, choroid plexus stromal cells, and choroid plexus epithelial cells produce CCL2, CXCL1, CXCL2, and IL-6 at 4 h after LPS injection (*red arrows*). The receptors for these cytokines were expressed by astrocytic endfeet that are located in close apposition to vascular endothelial cells and leptomeningeal cells. Thus, cytokine-mediated cell–cell interactions occur between the endothelial cells and perivascular astrocytic endfeet, and between the leptomeningeal stromal cells and subpial astrocytic endfeet. In addition, cytokines produced by choroid plexus stromal cells and epithelial cells may flow into the cerebrospinal fluid, and those produced by choroid plexus stromal cells may also flow into the brain parenchyma via the attachments of choroid plexus (*red arrows*). Thereafter, astrocytes produce CCL11 and CXCL10 at 4 and 24 h, and G-CSF at 24 h after LPS injection (*black arrows*). An initial step to activate stromal cells in the leptomeninges and choroid plexus, and in choroid plexus epithelial cells, may be triggered by nearby myeloid cells, although this remains to be proved (*black arrows with dotted line*).

CXCL1 was produced by vascular endothelial cells and CXCR2, a receptor for CXCL1proved to be expressed on astrocytic perivascular endfeet. These results were not surprising, given that the cell–cell interactions with cytokines as mediators occur between cells located close to each other and that vascular endothelial cells are in close apposition to perivascular astrocytic endfeet covering most of the perivascular surface of cerebral microcirculation (Fig. 7). Similarly, among CCL2, CXCL1, CXCL2, IL-6, and CXCL10 produced by leptomeningeal stromal cells, the receptors for CCL2, CXCL1, CXCL2, and IL-6 proved to be expressed on subpial astrocytic endfeet, which is also reasonable given the close apposition between leptomeningeal cells and subpial astrocytic endfeet that line the entire brain surface (Fig. 7).

In addition to the cell–cell interactions, choroid plexus epithelial cells may release cytokines into the ventricular space and choroid plexus stromal cells may release cytokines into the subarachnoid space, both of which result in the flow of cytokines into cerebrospinal fluid (Fig. 7). Recent studies revealed that cerebrospinal fluid has access to brain parenchymal interstitium and exchanges molecules with interstitial fluid^39^,^40^. Fluorescent tracers with low and intermediate molecular weights (759 Da and 3 kDa, respectively) in subarachnoid cerebrospinal fluid infiltrate the brain parenchyma along the paravascular spaces surrounding cortical penetrating arteries, whereas larger molecular weight fluorescent tracers (2000 kDa) do not^39^. Because molecular weights of CCL2, CXCL1, CXCL2, and CXCL10 are approximately 7–8 kDa and that of IL-6 is approximately 18 kDa, these cytokines presumably infiltrate the brain interstitium once they enter the subarachnoid space.

Another possible flow pathway for cytokines from the choroid plexus into the hippocampus involves the attachments of choroid plexus^24^, which consist of the ependyma, pia, and extremely thinned-out brain parenchyma between them, where the brain parenchyma and choroid plexus stroma are contiguous with no barrier (Fig. 7)^23^. Given that the hippocampus holds the attachments of choroid plexus at the fimbria, cytokines released by choroid plexus stromal cells may flow into the fimbria through the attachments of choroid plexus.

It is known that IL-1β expression is induced in the brain after systemic LPS administration. Although there was no increase in the hippocampal concentration of IL-1β up to 24 h after LPS injection in the present study, IL-1β was immunohistologically expressed by myeloid cells located in the leptomeninges and choroid plexus stroma and by epiplexus cells (myeloid cells on the luminal surface of choroid plexus epithelium^41^ at 1 and 4 h after LPS injection (Supplementary Fig. 5). Colocalization analysis following double immunofluorescence staining for IL-1β and Iba-1 indicated that the Pearson’s correlation coefficient ranged from 0.893 to 0.985 with the mean being 0.960 (11 fields), which was interpreted as a very high correlation between IL-1β- and Iba-1-immunofluorescence. These data are partially consistent with previous studies. Brain level of IL-1β was not increased by 16 h after a single i.p. injection of LPS (3 mg/kg) into mice, and increased at 28 h after LPS injection^18^. IL-1β protein expression was increased in macrophage-like cells in the leptomeninges and choroid plexus and microglia-like cells in the CVOs at 1.5, 4, and 8 h after i.p. or intravenous (i.v.) injection of LPS (2.5 mg/kg) into rats^12^. IL-1β mRNA expression appeared in the leptomeninges, choroid plexus, blood vessels, and CVOs at 0.5 and 2 h after i.p. injection of LPS (2.5 mg/kg) into rats^42^. IL-1β mRNA expression was increased in the striatum/thalamus but not in the hippocampus 3 h after i.v. injection of LPS (25 μg/mouse)^11^, whereas it was increased in the hypothalamus and hippocampus 1 and 2 h after i.p. injection of LPS (10 μg/mouse)^13^. Discrepancies in these studies may arise from differences in the dose, route, and timing of LPS injection.

As a possible role for endotoxemia-induced IL-1β-producing myeloid cells in the leptomeninges and choroid plexus, they may function as triggers for the activation of leptomeningeal stromal cells, choroid plexus stromal cells, and choroid plexus epithelial cells to produce cytokines via IL-1β–IL-1R signaling, since IL-1R1 was expressed by these cells. It has been reported that toll-like receptor 4 (TLR4) and CD14 are constitutively expressed in the leptomeninges and choroid plexus as well as CVOs^43^. The expression of CD14 mRNA in these regions is increased by systemic LPS challenge. There is no mRNA transcript for TLR4 or CD14 in the brain parenchyma except in limited brainstem regions^43^. Therefore, some of the hippocampal responses to systemic LPS challenge may originate from myeloid cells in the leptomeninges and choroid plexus, whereas others may originate from the activation of vascular endothelial cells by multiple inflammatory factors in circulating blood^44^(Fig. 7).

Microglia are known to be activated by systemic LPS administration^45^. Intravenous injection of LPS (1–5 mg/kg) into rats induces a morphological transition of microglia to macrophage-like cells in the hypothalamus, thalamus, and brainstem 8–24 h after LPS injection^46^. Microglia/brain macrophages collected by flow cytometric cell sorting from mice after i.p. injection of LPS (2 mg/kg) produce proinflammatory cytokines^47^. We did not recognize hippocampal microglia as a source of producing cytokines either at 4 or 24 h after LPS injection (3 mg/kg) by immunohistological staining. This may suggest that cytokines potentially produced by microglia were relatively small in amount until 24 h after LPS injection in this particular area. Our results are not inconsistent with those of a previous study^48^ in which microglial activation was not detectable at 24 h after a single i.p. injection of LPS (1 mg/kg) into mice. In their paradigm with daily LPS injections for 4 consecutive days, microglial activation was detectable 24 h after two and four LPS injections. Hippocampal microglia may be activated later than 24 h after LPS injection in our model.

In conclusion, we identified cells and cytokines in the brain that respond to endotoxin-induced systemic inflammation, whereby multiple cytokine levels were increased in hippocampal tissue. CCL2, CXCL1, CXCL2, and IL-6 were increased at 4 h after LPS injection; CCL11 and CXCL10 were increased at 4 and 24 h; and G-CSF was increased at 24 h. Cytokine concentrations increased at 4 h were expressed by leptomeningeal stromal cells, choroid plexus stromal cells, choroid plexus epithelial cells, and hippocampal vascular endothelial cells, all of which are located at the brain–immune interface. Receptors for the cytokines increased at 4 h were expressed by astrocytic endfeet. Cytokine concentrations increased at 24 h were expressed by astrocytes. Thus, cells of the brain–immune interface respond to endotoxemia with cytokine-mediated signals earlier than hippocampal parenchymal cells. In the parenchyma, astrocytes play a key role in responding to the signals by using endfeet located in close apposition to the interface cells via cytokine receptors.

## Methods

### Animals

Male C57BL/6N (B6) mice at the age of 3 months (body weight 21–32 g) were purchased from Japan SLC (Hamamatsu, Japan). Mice were intraperitoneally (i.p.) injected with LPS from *Escherichia coli* O55:B5 (Sigma, St Louis, MO, USA) dissolved in physiological saline at a dose of 3 mg/kg or with saline in a total volume of 7.5 mL/kg, and examined at 1, 4, and 24 h after injection. All mice were handled in accordance with the guidelines of Aichi Human Service Center and the Guide for the Care and Use of Laboratory Animals (National Academy Press, Washington, DC, USA). All experiments were approved by the Institutional Animal Care and Use Committee of Aichi Human Service Center.

### Tissue collection and protein extraction

Mice were anesthetized with sodium pentobarbital, and blood was removed from the right cardiac ventricle (1.0–1.2 mL/mouse) before tissues were sampled to reduce the contribution by cytokines contained in circulating blood. Spleens were harvested and cut into three pieces. After brains were harvested rapidly, both sides of the hippocampus were dissected out on ice under a stereoscopic microscope. A piece of spleen and one side of the hippocampus were separately homogenized using BioMasher II (Nippi, Tokyo, Japan) in 20 volumes of Tissue Protein Extraction Reagent (T-PER; Thermo Fisher Scientific, Waltham, MA, USA) containing Halt Protease Inhibitor Cocktail, EDTA-free (Thermo Fisher Scientific). Tissue homogenates were centrifuged at 13,000 rpm for 5 min at 4 °C, and supernatants were collected and snap frozen in liquid nitrogen.

### An initial pilot study: rationale for selecting 15 cytokines for multiplex assays

For an initial pilot study before determining tissue concentrations in the major study, we prepared protein extracts from the spleens and hippocampi of treated mice 1 and 4 h after LPS injection (*n* = 2 each) and naive mice (*n* = 5). We measured tissue concentrations of 33 cytokines (IL-1α, IL-1β, IL-2, IL-3, IL-4, IL-5, IL-6, IL-7, IL-9, IL-10, IL-12 [p40], IL-12 [p70], IL-13, IL-15, IL-17, TNF-α, granulocyte-colony stimulating factor [G-CSF], granulocyte and macrophage-CSF, macrophage-CSF, interferon [IFN]-γ, CXC-motif ligand [CXCL]1, CXCL2, CXCL5, CXCL9, CXCL10, CC-motif ligand [CCL]2, CCL3, CCL4, CCL5, CCL11, leukemia inhibitory factor [LIF], vascular endothelial growth factor, and CX3C-motif ligand [CX3CL]1) using MILLIPLEX MAP Mouse Cytokine/Chemokine Magnetic Bead Panel I (MCYTOMAG-70K, to measure concentrations of maximum 32 analytes) and Panel II (MCYP2MAG-73K, to measure CX3CL1 concentration) (Merck Millipore, Billerica, MA, USA). Fluorescent signals were measured with Luminex 200 with xPONENT software (Luminex, Austin, TX, USA), and the median fluorescence intensity was obtained. Raw data were analyzed with MasterPlex QT software (Hitachi Solutions–MiraiBio Group, South San Francisco, CA, USA) using a best-fit method for calculating cytokine concentrations of samples. All samples were run in duplicate.

### The major study: multiplex cytokine assays

The initial pilot study suggested that of 33 cytokines, 15 (CCL2, CCL3, CCL4, CCL11, CXCL1, CXCL2, CXCL9, CXCL10, G-CSF, IL-1β, IL-6, IL-10, IFN-γ, LIF, and TNF-α) were present at measurable levels in the intact splenic and hippocampal tissue extracts, and the concentrations of these cytokines tended to increase in the spleens of treated mice compared with naive mice. Therefore, we decided to determine the concentrations of these cytokines for the major study using MCYTOMAG-70K-15. In the major study, we prepared protein extracts from the spleens and hippocampi of mice at 1, 4, and 24 h after LPS injection (*n* = 5 per experimental condition) and after saline injection (saline control; *n* = 7 in total; see Statistics). We designed each experiment so that one or two mice from all conditions (LPS 1 h, LPS 4 h, LPS 24 h and saline) were examined at the same time. We performed thus designed experiments 4 times on different days. Details of measuring fluorescent signals and analyzing data were the same as in the initial pilot study. All samples were run in duplicate. Data are shown as the amount of cytokines per wet weight of tissue.

### Histological preparation and immunohistological staining for cytokines

For histological and immunohistological examinations, sections were prepared from mice at 1, 4, and 24 h after LPS injection and compared with control sections prepared from mice at 1 h after saline injection (saline control). The total number of mice examined was 53: *n* = 16 for saline control and 4 h after LPS injection; *n* = 9 for 1 h after LPS injection; and *n* = 12 for 24 h after LPS injection. Mice were anesthetized with sodium pentobarbital, perfused transcardially with phosphate-buffered saline (PBS) to remove blood, and perfused with 100 mL of 4% paraformaldehyde at a rate of 8 mL/min. Heads were placed in the same fixative at 4 °C overnight. Brains were removed, cryo-protected with 30% sucrose, embedded in cryo-embedding compound (Super Cryo-embedding Medium; Section-lab, Hiroshima, Japan), and frozen with normal hexane cooled with dry ice. Frozen tissue blocks were set for coronal sectioning using cryostats (Bright 5030 microtome; Bright Instruments, Cambridgeshire, UK and CM1950; Leica, Wetzlar, Germany). Immediately before making a section, the surface of a frozen block was sealed every time with CryoFilm (Section-lab), a transparent adhesive film^49^, and sectioned at 8-μm thickness. Frozen sections attached to film were dried in a cryostat for 30 min, treated with 100% ethanol for 1 min, and stored in PBS at 4 °C.

For immunohistochemical staining, sections on the film were pretreated with 0.3% H_2_O_2_ in PBS to block endogenous peroxidase activity, and with 5% bovine serum albumin in PBS to block non-specific binding sites. Sections on film were incubated with primary antibodies at 4 °C for 2 days or at room temperature overnight. Details of the primary antibodies are listed in Table 2. Sections on film were then incubated with reagents from the ImmPRESS HRP Anti-Goat, Anti-Rabbit, or Anti-Rat IgG (Peroxidase) Polymer Detection Kit (Vector Laboratories, Burlingame, CA, USA), in accordance with the species origin of primary antibodies, at room temperature for 1 h. Reactions were visualized by incubating sections with 0.4 mg/mL 3,3′-diaminobenzidine (DAB) in PBS containing 0.006% H_2_O_2_ for 3–30 min. Sections on film were cleared with 25, 50, 75, and 100% ethanol sequentially, mounted on slide glass, and coverslipped with mounting medium (Super Cryo-Mounting Medium; Section-lab), followed by ultraviolet radiation to solidify the mounting medium at room temperature for 15 min. Sections were examined with Eclipse E600 and 80i light microscopes (Nikon, Tokyo, Japan). The Eclipse 80i was equipped with a digital camera control unit, DS-Fi2/DS-L3 (Nikon).

For double immunofluorescence staining, frozen sections on the film were incubated with primary antibodies at 4 °C for 2 days or at room temperature overnight. Combinations of antibodies are shown in Table 3. Sections were then incubated with Alexa Fluor 568- or 488-conjugated anti-goat, anti-rabbit, or anti-rat IgG secondary antibodies (Invitrogen, Burlington, ON, Canada), in accordance with the species origin of primary antibodies, at 4 °C overnight. Nuclei were counterstained with 4′,6-diamidino-2-phenylindole (DAPI). Sections on film were mounted on slide glass and coverslipped with mounting medium (Fluorescence Mounting Medium; DAKO, Glostrup, Denmark). Sections were observed with a confocal laser scanning microscope (FLUOVIEW FV1000; Olympus, Tokyo, Japan).

Colocalization analyses of double immunofluorescence images were performed according to a guide reported previously^50^. For every combination of primary antibodies (Table 3), 5 to 11 (with the mean being 7.7) visual fields per experimental condition were examined using Image-Pro Plus (Media Cybernetics, Rockville, MD, USA). A visual field consisted of a square with a side length of 200 μm. In each field, the “Colocalization Analysis” command was executed on a merged fluorescence image with the setting of correlation between red and green components. Regions of interest were drawn to cover individual cells over the image. Scatterplots were made and Pearson’s correlation coefficients were measured from individual regions of interest, and the mean coefficient represented the field. The size of a correlation coefficient was interpreted as previously reported^51^.

Reliability of antibodies was confirmed in four ways. First, the specificity was validated based on the fact that omission of primary antibodies abolished staining. Second, pre-absorption of antibodies with immunizing peptides abolished staining (Supplementary Methods, Supplementary Table 3, and Supplementary Fig. 6). Third, isotype immunoglobulins did not produce staining (Supplementary Methods, Supplementary Table 4, and Supplementary Fig. 7). Fourth, we performed immunohistological staining also with splenic sections from LPS-treated mice and saline controls to compare time-dependent changes in the immunoreactivity with the results from multiplex cytokine assays of the spleen, and confirmed the consistency (Supplementary Methods and Supplementary Figs 8 and 9).

### Immunohistochemistry for vimentin and measurement of vimentin-immunopositive areas in hippocampal sections

We quantified the intensity of vimentin immunoreactivity in hippocampal sections as a marker for astrocytic reaction. Sections on film containing the hippocampus were immunohistochemically stained with an anti-vimentin antibody (Table 2) and visualized with DAB. These sections were used to measure the area of vimentin-immunopositive structures. The area of interest was a rectangle 330 μm wide and 440 μm deep that was set on the hippocampus perpendicular to the direction of the stratum pyramidale so that it covered the strata lacunosum-moleculare, radiatum, pyramidale, and oriens. The total vimentin-immunopositive area within the area of interest was automatically detected and measured using Image-Pro Plus (Media Cybernetics, Rockville, MD, USA). Six areas of interest were set on three consecutive hippocampal sections per mouse, and the mean of six measurements represented each individual mouse. Mean vimentin-immunopositive area was divided by the area of interest, and the percentage was calculated.

### Statistics

Because concentrations of cytokines determined in mice at 1, 4, and 24 h after saline injection were similar for all cytokines examined, the three experimental conditions were combined as a single “saline control” (*n* = 7). Tissue cytokine concentrations in mice from the four experimental conditions (saline control and 1, 4, and 24 h after LPS injection) were analyzed using one-way analysis of variance (ANOVA; single main effect of treatment) with non-repeated measures in the spleen and hippocampus. Values of *p < *0.05 were considered significant. For each cytokine, if the main effect of treatment was significant, mean concentrations were compared among the four experimental conditions with post hoc tests using Tukey’s procedure.

Percentages of the vimentin-immunopositive area in mice from three experimental conditions (saline control, 4 h, and 24 h after LPS injection) were analyzed using one-way ANOVA with non-repeated measures. Mean percentages were compared among the three experimental conditions with post hoc tests using Tukey’s procedure.

## Additional Information

**How to cite this article**: Hasegawa-Ishii, S. *et al.* Endotoxemia-induced cytokine-mediated responses of hippocampal astrocytes transmitted by cells of the brain–immune interface. *Sci. Rep.* **6**, 25457; doi: 10.1038/srep25457 (2016).

## Supplementary Material

Supplementary Information

## Figures and Tables

**Table 1:** Criteria for grouping cytokines.

Group	Criteria	Cytokines		Graph Color in Figs 1 and 2
	Tissue cytokine concentration	Spleen	Hippocampus	
A	increased at 1 h and returned to the control level at 4 h after LPS injection	IL-10	TNFα	pink
		TNFα		
B	increased at 1 h, remained elevated at 4 h and returned to the control level at 24 h after LPS injection	CCL2		yellow
		CCL3		
		CCL4		
		CXCL1		
		CXCL2		
		CXCL10		
		IL-1β		
		IL-6		
		LIF		
C	increased at 4 h and returned to the control level at 24 h after LPS injection	CCL11IFNγ	CCL2	blue
			CXCL1	
			CXCL2	
			CXCL9	
			IL-6	
			LIF	
D	increased at 4 h, remained elevated at 24 h after LPS injection	G-CSF	CCL11	orange
			CXCL10	
E	increased at 24 h after LPS injection		G-CSF	green
F	did not increase at any time after LPS injection	CXCL9	CCL3	white
			CCL4	
			IFNγ	
			IL-1β	
			IL-10	

Note: The determination of the changes in the tissue cytokine concentration was based on the results of Tukey’s post-hoc tests indicating that differences between experimental groups were significant or showed a trend toward significance.

**Table 2:** List of antibodies.

Antibody against	Host	Dilution	Source
CCL2	Goat	50	R&D Systems (Minneapolis, MN, USA)
CCL11	Goat	200	R&D Systems
CCR2	Goat	100	Novus Biologicals (Cambridge, UK)
CD31	Rat	200	BMA Biomedicals (Augst, Switzerland)
CXCL1	Goat	100	R&D Systems
CXCL2	Rabbit	100	AbD Serotec, Bio-Rad Laboratories (Hercules, CA, USA)
CXCL10	Goat	50	R&D Systems
CXCR2	Rat	50	R&D Systems
ER-TR7	Rat	200	BMA Biomedicals
G-CSF	Goat	200	SantaCruz (Dallas, TX, USA)
GFAP	Rabbit	1000	Dako Cytomation (Glostrup, Denmark)
Iba-1	Rabbit	1000	Wako (Osaka, JAPAN)
IL-1β	Goat	200	R&D Systems
IL-1R1	Goat	20	R&D Systems
IL-6	Goat	50	R&D Systems
IL-6	Rabbit	100	Cell Signaling Technology (Danvers, MA, USA)
IL-6R	Rat	100	R&D Systems
MyD88	Goat	500	R&D Systems
NG2	Rabbit	200	Millipore (Tomecula, CA, USA)
S100	Rabbit	100	Dako Cytomation
Vimentin	Rabbit	200	Cell Signaling Technology

G-CSF, granulocyte-colony stimulating factor; GFAP, Glial fibrillary acidic protein; Iba-1, Ionized-calcium binding adaptor molecule-1; MyD88, myeloid differentiation primary response 88.

**Table 3:** Combinations of primary antibodies for double immunofluorescence staining.

Antibodies for cytokines (host)	Antibodies for cell markers (host)
CCL2 (goat)	CD31 (rat)
CCL2 (goat)	ER-TR7 (rat)
CCL2 (goat)	Iba-1 (rabbit)
CCL2 (goat)	NG2 (rabbit)
CCL2 (goat)	S100 (rabbit)
CCL11 (goat)	GFAP (rabbit)
CCR2 (goat)	GFAP (rabbit)
CXCL1 (goat)	CD31 (rat)
CXCL1 (goat)	ER-TR7 (rat)
CXCL1 (goat)	Iba-1 (rabbit)
CXCL1 (goat)	NG2 (rabbit)
CXCL1 (goat)	S100 (rabbit)
CXCL2 (rabbit)	CD31 (rat)
CXCL2 (rabbit)	CXCL1 (goat)
CXCL10 (goat)	CD31 (rat)
CXCL10 (goat)	GFAP (rabbit)
CXCR2 (rat)	GFAP (rabbit)
G-CSF (goat)	GFAP (rabbit)
IL-1β (goat)	Iba-1 (rabbit)
IL-6 (goat)	CD31 (rat)
IL-6 (goat)	Iba-1 (rabbit)
IL-6 (goat)	NG2 (rabbit)
IL-6 (goat)	S100 (rabbit)
IL-6 (rabbit)	CD31 (rat)
IL-6 (rabbit)	ER-TR-7 (rat)
IL-6R (rat)	GFAP (rabbit)
