# Supplementary material for: Endotoxemia-induced cytokine-mediated responses of hippocampal astrocytes transmitted by cells of the brain–immune interface
Source: Sci Rep. 2016 May 5;6:25457. doi: 10.1038/srep25457 (PMC4857737; doi:10.1038/srep25457)
Supplement: Supplementary Information [file srep25457-s1.pdf]

Supplementary Materials

Supplementary Methods
Supplementary Tables (4 tables)
Supplementary Figures (9 figures)

Endotoxemia-induced cytokine-mediated responses of hippocampal astrocytes transmitted by cells of the brain–immune interface

Sanae Hasegawa-Ishii[1,2,3], Muneo Inaba[4], Hiroyuki Umegaki[5], Keiko Unno[6], Keiji Wakabayashi[3] and Atsuyoshi Shimada*[2]

[1] Japan Society for the Promotion of Science, 5-3-1 Kojimachi, Chiyoda, Tokyo 102-0083, Japan
[2] Department of Pathology and Laboratory Medicine, Central Hospital, Aichi Human Service Center, 713-8 Kamiya, Kasugai, Aichi 480-0392, Japan
[3] Graduate school of Integrated Pharmaceutical and Nutritional Sciences, University of Shizuoka, 52-1 Yada, Suruga-ku, Shizuoka 422-8526, Japan
[4] First Department of Internal Medicine, Kansai Medical University, 2-5-1 Shinmachi, Hirakata, Osaka 573-1010, Japan
[5] Department of Geriatrics, Graduate School of Medicine, Nagoya University, 65 Tsurumai, Showa-ku, Nagoya, Aichi 466-8550, Japan
[6] Department of Neurophysiology, School of Pharmaceutical Sciences, University of Shizuoka, 52-1 Yada, Suruga-ku, Shizuoka 422-8526, Japan

*Corresponding author: Atsuyoshi Shimada, M.D., Ph.D.
Department of Pathology and Laboratory Medicine, Central Hospital, Aichi Human Service Center
713-8 Kamiya, Kasugai, Aichi 480-0392, Japan
Tel: +81-0568-88-0811 (ext. 3521); Fax: +81-0568-88-0829
E-mail: ats7@inst-hsc.jp

Supplementary Methods

Validation of antibodies for immunohistological studies

1. Omission of primary antibodies (negative control)

In the immunohistochemical procedures, the primary antibodies were replaced with dilution buffer (5 % bovine serum albumin in phosphate-buffered saline), keeping all the other steps the same. We confirmed that the omission of primary antibodies completely abolished the immunostaining for all antibodies listed in Table 2 (data not shown).

2. Pre-absorption of primary antibodies (pre-absorption control)

Primary antibodies (for CCL2, CXCL1, CXCL2, IL-6, CCL11, CXCL10, G-CSF, IL-6R, and CCR2) were incubated with 20- or 40-fold molar excess of corresponding immunizing peptides (as listed in Supplementary Table 3) for 4 °C overnight, centrifuged at 13,000 rpm for 15 min at 4 °C, and supernatants collected (pre-absorbed antibodies). Pre-absorbed antibodies and non-treated antibodies were used in the same sets of experiments in which adjacent sections prepared from the same mice were stained immunohistochemically. We confirmed that the pre-absorption of primary antibodies abolished the immunostaining for all antibodies examined (Supplementary Fig. 6).

3. Replacement of primary antibodies with isotype immunoglobulins (isotype immunoglobulin control)

In the immunohistochemical procedures, the primary antibodies were replaced with isotype immunglobulins, keeping all the other steps the same. As listed in Table 2, hosts of the primary antibodies were rat, rabbit, and goat. Accordingly, primary antibodies were replaced with non-immunized rat IgG, non-immunized rabbit IgG, and non-immunized goat IgG prepared at the comparable concentrations of primary antibodies (Supplementary Table 4). We confirmed that these isotype IgGs did not produce staining (Supplementary Fig. 7).

4. Immunohistological staining with spleen sections

We performed immunohistochemical and immunofluorescence staining using splenic sections from LPS-treated mice and saline control. The spleen contains immune cells that produce cytokines in response to intraperitoneally injected LPS. As shown in Results, LPS-induced elevation in the tissue cytokine concentration was much higher in the spleen than in the hippocampus. Therefore, we compared the time-dependent changes in the immunoreactivity with the results from multiplex cytokine assays of the spleen. We confirmed that the particular splenic cell populations were individually immunolabeled for CCL2, CXCL1, CXCL2, IL-6, CCL11, CXCL10, and G-CSF. In addition, the time-dependent changes in the staining intensity were generally consistent with the patterns obtained from the multiplex cytokine assays (Supplementary Figs. 8 and 9).

Supplementary Table 1

Results of one-way ANOVA on changes in the splenic tissue cytokine concentrations after systemic LPS challenge

**Cytokine**	**F value**	**p value**
IL-10	$F(3, 18) = 8.26137$	$p = 0.001147$
TNFα	$F(3, 18) = 103.8118$	$p < 0.000001$
CCL2	$F(3, 16) = 53.8047$	$p < 0.000001$
CCL3	$F(3, 18) = 22.44866$	$p = 0.000003$
CCL4	$F(3, 18) = 21.21379$	$p = 0.000004$
CXCL1	$F(3, 18) = 137.2857$	$p < 0.000001$
CXCL2	$F(3, 18) = 256.8819$	$p < 0.000001$
CXCL10	$F(3, 18) = 49.2289$	$p < 0.000001$
IL-1β	$F(3, 16) = 6.92975$	$p = 0.003345$
IL-6	$F(3, 18) = 26.65326$	$p = 0.000001$
LIF	$F(3, 16) = 18.69895$	$p = 0.000018$
CCL11	$F(3, 18) = 10.0069$	$p = 0.000419$
IFNγ	$F(3, 18) = 4.438745$	$p = 0.016742$
G-CSF	$F(3, 18) = 4.89689$	$p = 0.011630$

Supplementary Table 2

Results of one-way ANOVA on changes in the hippocampal tissue cytokine concentrations after systemic LPS challenge

**Cytokine**	**F value**	**p value**
TNFα	$F(3, 18) = 3.66731$	$p = 0.031933$
CCL2	$F(3, 16) = 8.90841$	$p = 0.001054$
CXCL1	$F(3, 18) = 10.73736$	$p = 0.000283$
CXCL2	$F(3, 18) = 7.48300$	$p = 0.001866$
CXCL9	$F(3, 18) = 10.23946$	$p = 0.000369$
IL-6	$F(3, 18) = 6.697020$	$p = 0.003137$
LIF	$F(3, 16) = 4.995981$	$p = 0.012391$
CCL11	$F(3, 18) = 28.5869$	$p < 0.000001$
CXCL10	$F(3, 18) = 56.0275$	$p < 0.000001$
G-CSF	$F(3, 18) = 3.89598$	$p = 0.026255$

Supplementary Table 3
List of peptides for pre-absorption of antibodies

Peptide	Molar ratio (antibody : peptide)	Source of peptide
Recombinant mouse CCL2	1 : 40	R&D Systems
Recombinant mouse CCL11	1 : 20	R&D Systems
Recombinant mouse CCR2	1 : 40	Custom synthesis<br>“SHSLFTRSIQELDEGATTPYDYDDGEPC”<br>Wako Pure Chemical Industries (Osaka, JAPAN)
Recombinant mouse CXCL1	1 : 40	R&D Systems
Recombinant mouse CXCL2	1 : 40	AbD Serotec
Recombinant mouse CXCL10	1 : 20	R&D Systems
Recombinant human G-CSF	1 : 20	SantaCruz
Recombinant mouse IL-6	1 : 20	R&D Systems
Recombinant mouse IL-6R	1 : 20	R&D Systems

Supplementary Table 4
List of isotype immunoglobulins for isotype control studies

Isotype immunoglobulin	Dilution	Final concentration	Source
Normal rat IgG	100	4 μg/mL	Santa Cruz, sc-2026
Normal rabbit IgG	200	2 μg/mL	Santa Cruz, sc-2027
Normal goat IgG	200	2 μg/mL	Santa Cruz, sc-2028

Supplementary Figure 1

Identification of CCL2-expressing cells in the choroid plexus stroma following systemic LPS challenge

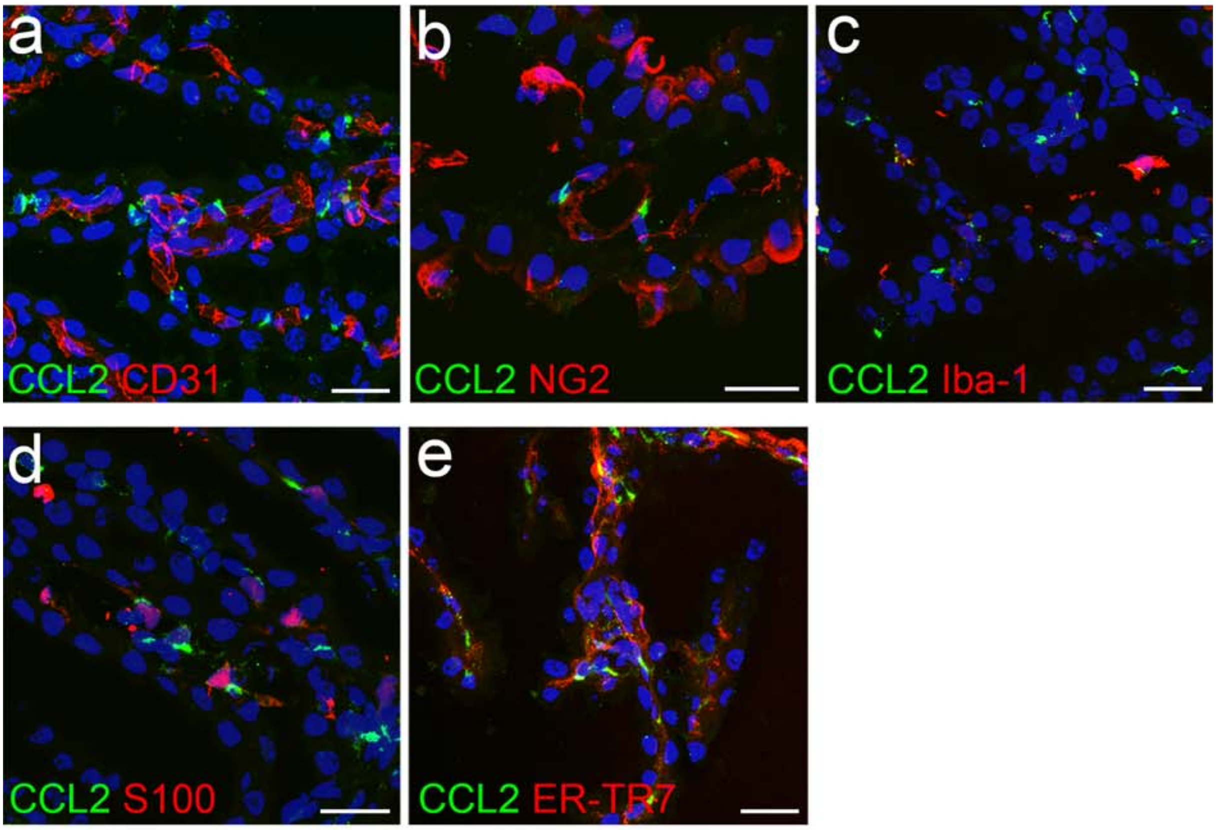

Double immunofluorescence study revealed that CCL2-expressing cells in the choroid plexus stroma did not exhibit cell markers such as CD31 (a), NG2 (b), Iba-1 (c), S100 (d), or ER-TR7 (e), indicating that the major population of CCL2-expressing cells in the choroid plexus stroma were not endothelial cells, pericytes, myeloid cells, arachnoid cells, or fibroblasts, respectively. Nuclei were counterstained with DAPI. All photoimages were obtained from mice at 4 h after LPS injection. Scale bars 20 μm

Supplementary Figure 2

Identification of CCL2-expressing cells in the spleen following systemic LPS challenge

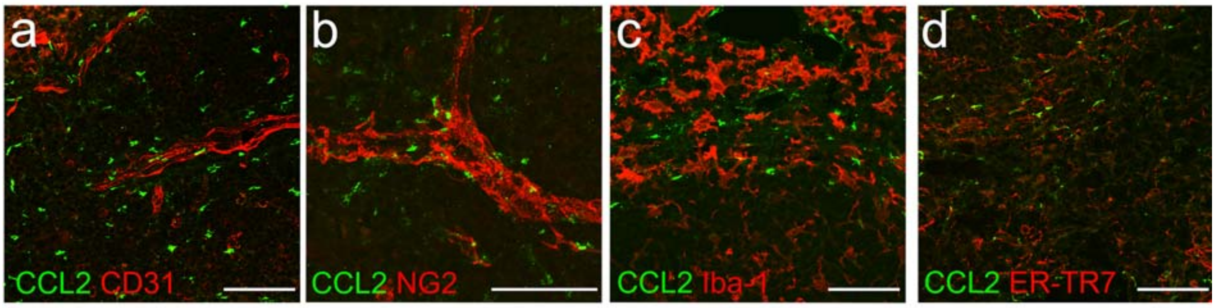

Double immunofluorescence study revealed that CCL2-expressing cells in the spleen did not exhibit cell markers such as CD31 (a), NG2 (b), Iba-1 (c), or ER-TR7 (d), indicating that the major population of CCL2-expressing cells in the spleen was not endothelial cells, pericytes, myeloid cells, or fibroblasts, respectively. Scale bars 50 μm

Supplementary Figure 3

CXCL1 and CXCL2 expression in the hippocampus following systemic LPS challenge

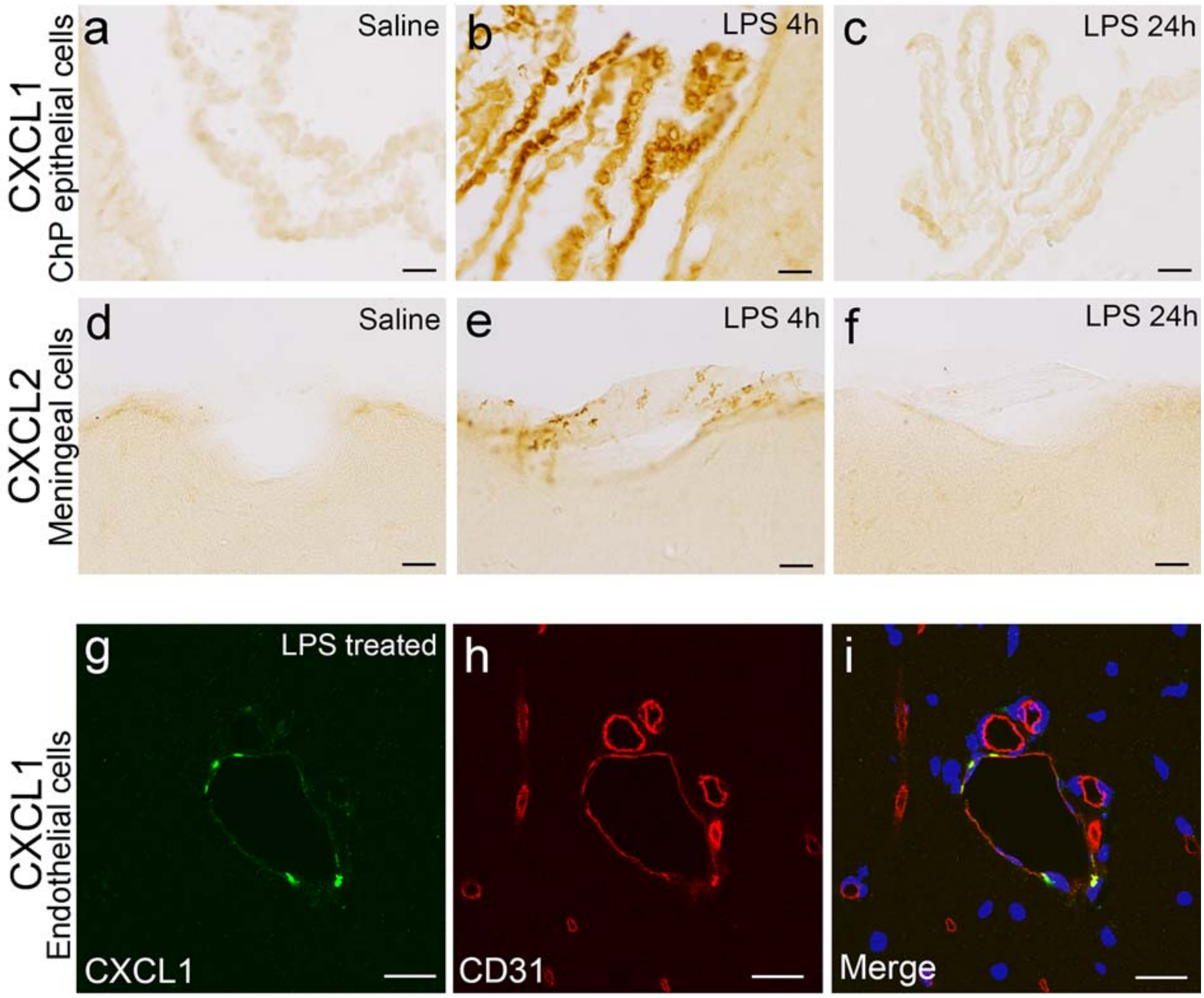

Immunohistochemistry revealed that CXCL1 expression was not detected in the saline control (a) and was increased in the choroid plexus epithelial cells 4 h after LPS injection (b). CXCL1 expression returned to the control appearance 24 h after LPS injection (c). Immunohistochemistry revealed that CXCL2 expression was not detected in the saline control (d) and was increased in the leptomeninges 4 h after LPS injection (e). CXCL2 expression returned to the control appearance 24 h after LPS injection (f). Double immunofluorescence study revealed that CXCL1-expressing cells along the hippocampal blood vessels were immunopositive for CD31, indicative of endothelial cells (g–i). Scale bars 20 μm

Supplementary Figure 4

Identification of IL-6-producing cells in the hippocampus following systemic LPS challenge

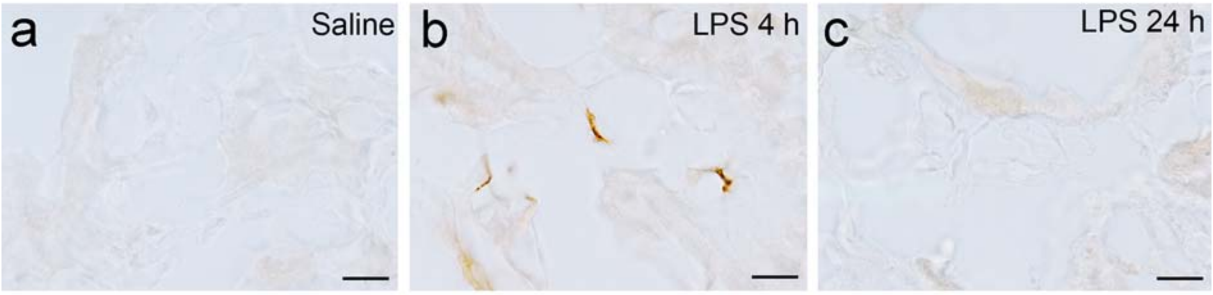

Immunohistochemistry revealed that IL-6 expression was not detected in the saline control (a) and was increased in the choroid plexus stroma 4 h after LPS injection (b). IL-6 expression returned to the control appearance 24 h after LPS injection (c). Scale bars 10 μm

Supplementary Figure 5

IL-1β expression in the choroid plexus following systemic LPS challenge

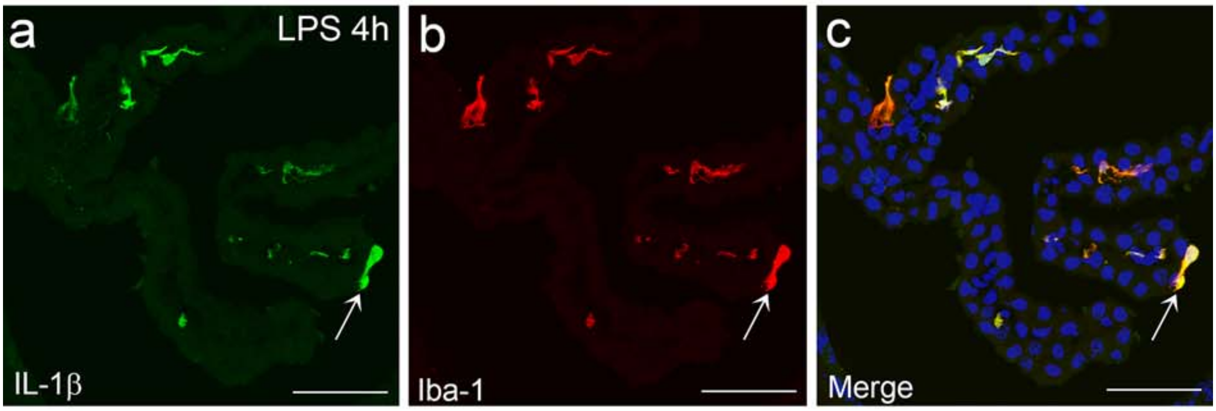

At 4 h after LPS injection, IL-1β-immunopositive myeloid cells in the choroid plexus expressed IL-1β. Many of them were located in the stroma. Some cells double immunopositive for IL-1β and Iba-1 were on the luminal surface of choroid plexus epithelium, indicative of epiplexus cells (white arrows). Scale bars 50 µm

Supplementary Figure 6

Pre-absorption control for immunohistochemistry

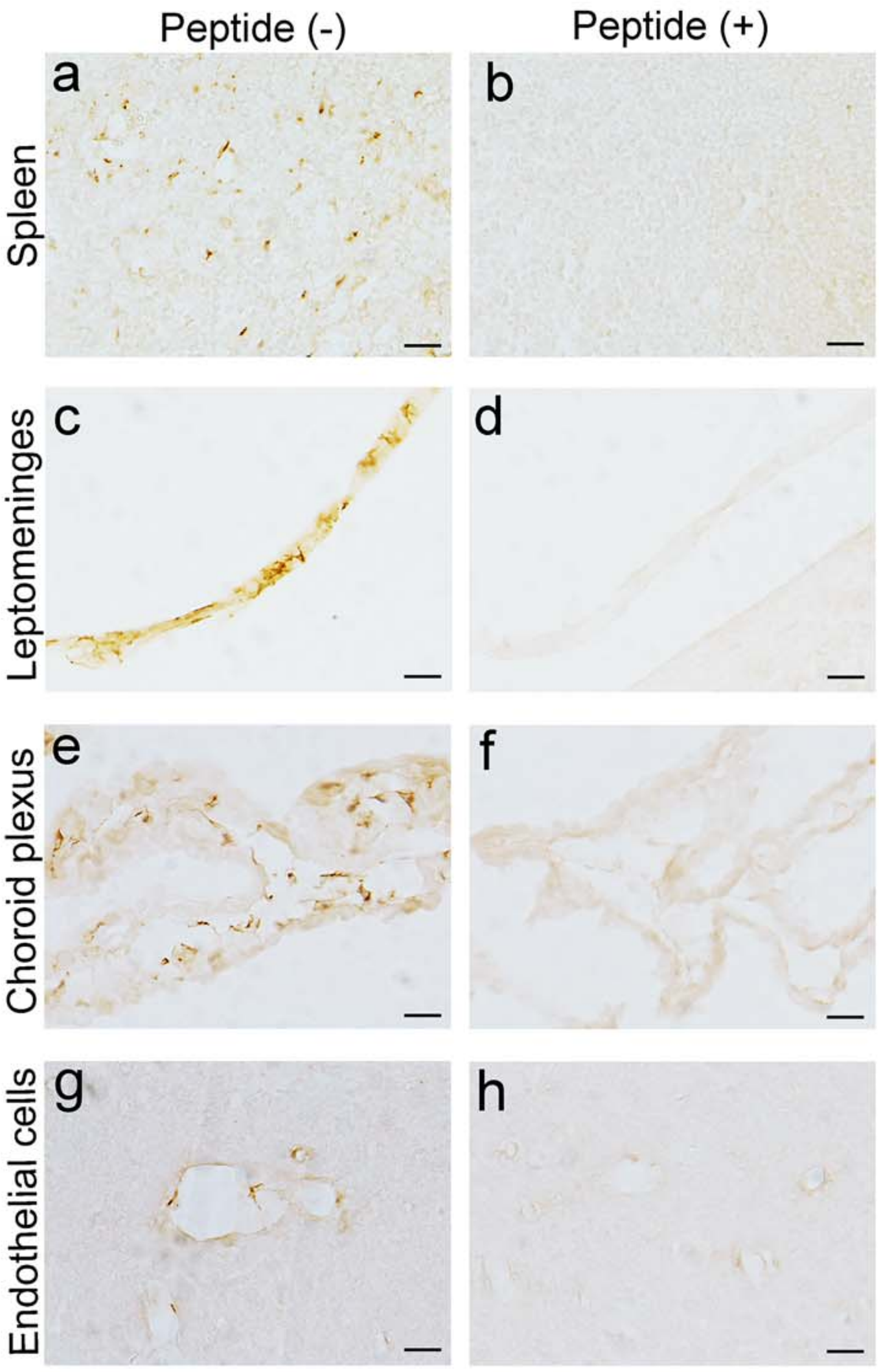

An anti-CCL2 antibody was incubated with 40-fold molar excess of immunizing peptide (pre-absorbed antibody preparation; Supplementary Methods). Sections of the spleen and hippocampus were prepared from mice at 4 h after LPS injection. In the sections for which the pre-absorbed antibody preparation was used during the immunohistochemical procedures [Peptide (+)], immunostaining was abolished from the spleen (b), leptomeninges (d), choroid plexus (f), and endothelial cells (h), compared with the sections for which non-treated antibodies were used [Peptide (−); a, c, e, and g].
Scale bars 20 μm

Supplementary Figure 7

Isotype immunoglobulin control for immunohistochemistry

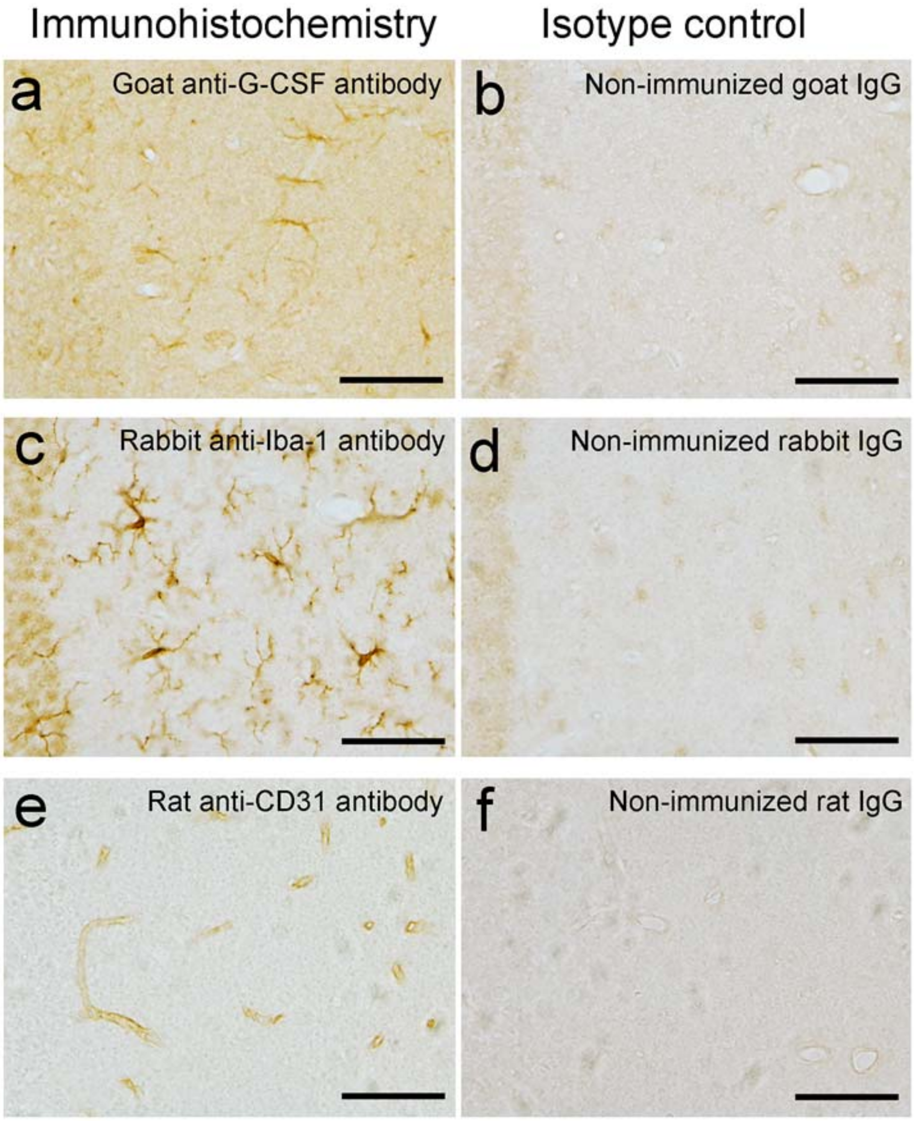

Hippocampal sections were prepared from mice at 24 h after LPS injection. In the sections for which non-immunized goat (b), rabbit (d), and rat (f) immunoglobulins were used during the immunohistochemical procedures (Isotype control), no staining was detected, compared with the sections for which goat anti-G-CSF antibody (a), rabbit anti-Iba-1 antibody (c), and rat anti-CD31 antibody (e) were used, respectively. Scale bars 50 μm

Supplementary Figure 8

CCL2, CXCL1, CXCL2, and IL-6 expression in the spleen following systemic LPS challenge

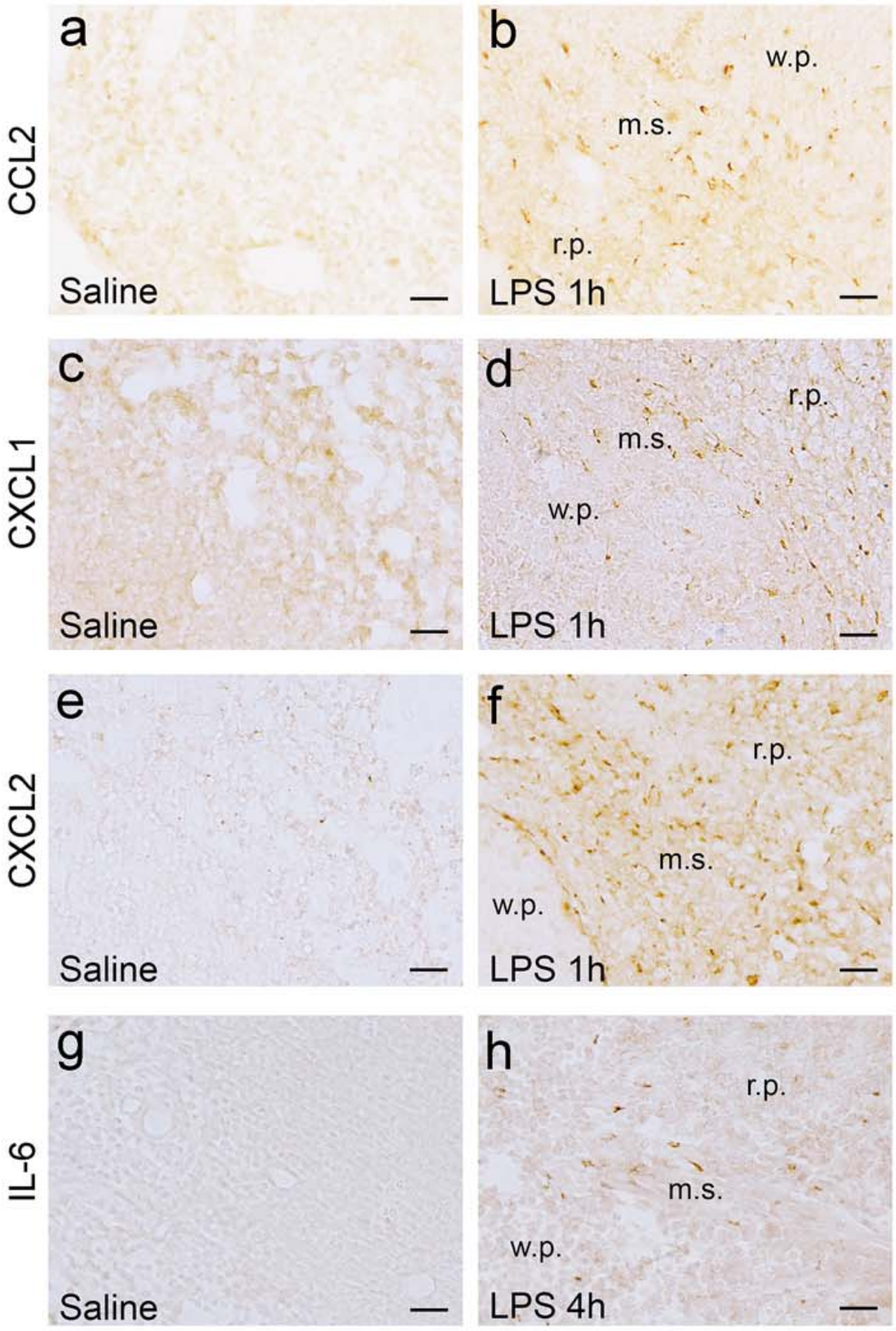

Immunohistochemistry revealed that CCL2 expression was not detected in any region of the spleen in the saline control (a) and was increased in the red pulp and marginal sinus of the spleen 1 h after LPS injection (b). CXCL1 expression was not detected in any region of the spleen in the saline control (c) and was increased in the red pulp and marginal sinus of the spleen 1 h after LPS injection (d). CXCL2 expression was not detected in any region of the spleen in the saline control (e) and was increased in the red pulp and marginal sinus of the spleen 1 h after LPS injection (f). IL-6 expression was not detected in any region of the spleen in the saline control (g) and was increased in the marginal sinus of the spleen 4 h after LPS injection (f). m.s., marginal sinus; r.p., red pulp; w.p., white pulp. Scale bars 20 μm

Supplementary Figure 9

CCL11, CXCL10, and G-CSF expression in the spleen following systemic LPS challenge

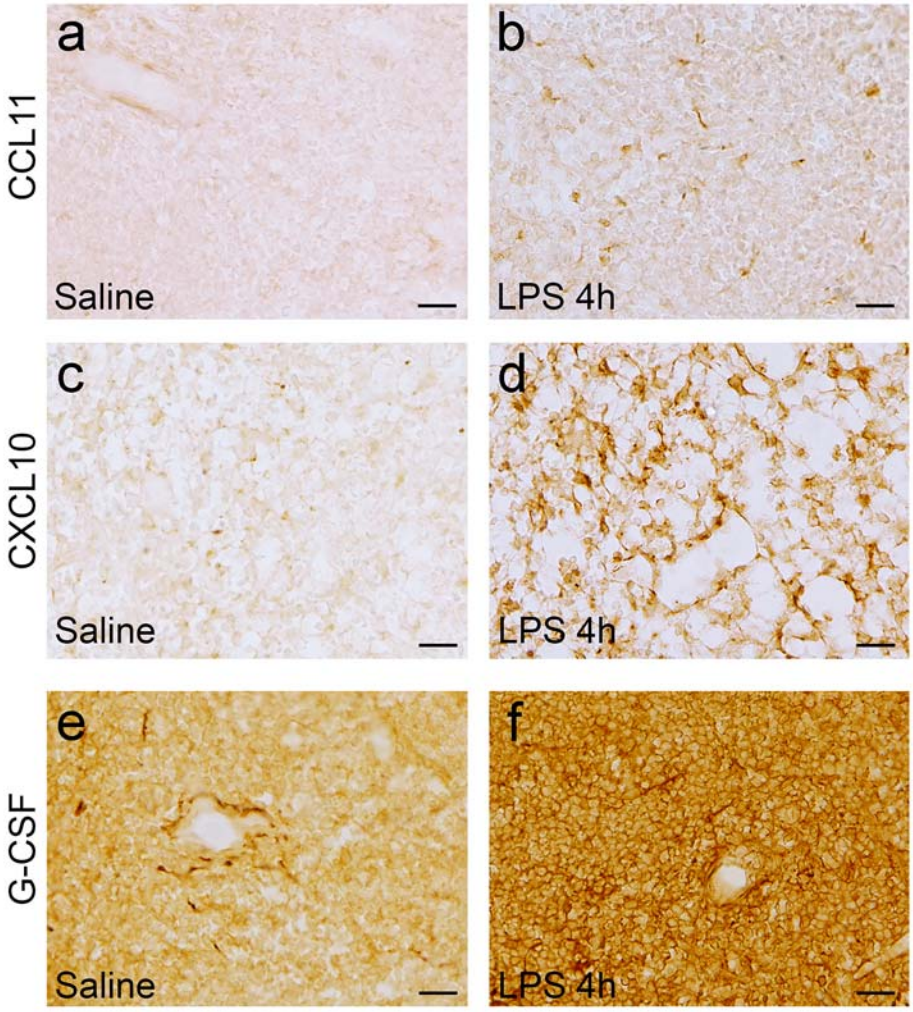

Immunohistochemistry revealed that CCL11 expression was rare in all regions of the spleen in the saline control (a) and was increased in the red pulp of the spleen 4 h after LPS injection (b). CXCL10 expression was almost not detected in any region of the spleen in the saline control (c) and was increased in the red pulp of the spleen 4 h after LPS injection (d). G-CSF expression was limited to around the blood vessels in the saline control (e) and was increased throughout the spleen 4 h after LPS injection (f). Scale bars 20 μm
